# In Situ Formed Tribofilms as Efficient Organic/Inorganic Hybrid Interlayers for Stabilizing Lithium Metal Anodes

**DOI:** 10.1007/s40820-023-01210-6

**Published:** 2023-10-24

**Authors:** Shaozhen Huang, Kecheng Long, Yuejiao Chen, Tuoya Naren, Piao Qing, Xiaobo Ji, Weifeng Wei, Zhibin Wu, Libao Chen

**Affiliations:** 1 State Key Laboratory of Powder Metallurgy, Central South University, Changsha, 410083 People’s Republic of China; 2 College of Chemistry and Chemical Engineering, Central South University, Changsha, 410083 People’s Republic of China

**Keywords:** Lithium metal anode, Organic/inorganic hybrid interlayers, High current density, Fluoropolymer grease, Local desolvation environment

## Abstract

The practical application of Li metal anodes (LMAs) is limited by uncontrolled dendrite growth and side reactions. Herein, we propose a new friction-induced strategy to produce high-performance thin Li anode (Li@CFO). By virtue of the in situ friction reaction between fluoropolymer grease and Li strips during rolling, a robust organic/inorganic hybrid interlayer (lithiophilic LiF/LiC_6_ framework hybridized -CF_2_-O-CF_2_- chains) was formed atop Li metal. The derived interface contributes to reversible Li plating/stripping behaviors by mitigating side reactions and decreasing the solvation degree at the interface. The Li@CFO||Li@CFO symmetrical cell exhibits a remarkable lifespan for 5,600 h (1.0 mA cm^−2^ and 1.0 mAh cm^−2^) and 1,350 cycles even at a harsh condition (18.0 mA cm^−2^ and 3.0 mAh cm^−2^). When paired with high-loading LiFePO_4_ cathodes, the full cell lasts over 450 cycles at 1C with a high-capacity retention of 99.9%. This work provides a new friction-induced strategy for producing high-performance thin LMAs.

**Supplementary Information:**

The online version contains supplementary material available at 10.1007/s40820-023-01210-6.

## Highlights

- The robust organic/inorganic hybrid interlayer derived from in situ formed tribofilms were fabricated by using a scalable rolling method.
- The interlayer facilitates dendrite-free lithium metal anodes by building local de-solvation environments near the interface and inhibiting both dendrite growth and electrolytes corrosion.
- The symmetrical cell exhibits a remarkable lifespan of 5,600 h (1.0 mA cm^-2^ and 1.0 mAh cm^-2^) and 1,350 cycles even at a harsh condition (18.0 mA cm^-2^ and 3.0 mAh cm^-2^).

**Supplementary Information:**

The online version contains supplementary material available at 10.1007/s40820-023-01210-6.

## Graphical abstract

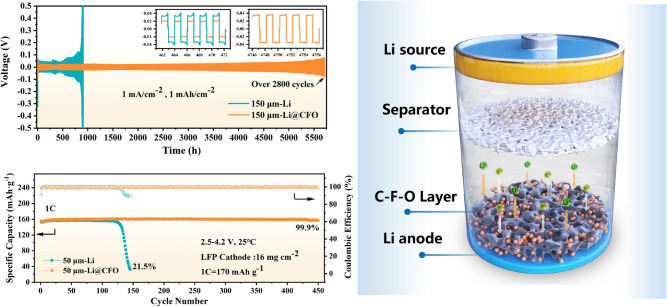

## Introduction

The energy density of commercial lithium-ion batteries (LIBs) is close to the theoretical limit and no longer satisfy the increasing need. Thus, lithium metal anodes (LMAs) with ultra-high theoretical specific capacity (3860 mAh g^−1^) and ultralow redox potential (−3.04 V vs. standard hydrogen electrode) has been regarded as a promising next-generation anode for rechargeable lithium metal batteries (LMBs) [1–4]. However, the Li dendrite growths, huge volume changes and undesirable side reactions with electrolytes hinder the commercial application of LMBs [5–11]. To solve the issues, great efforts have been introduced to improve the performance of LMAs. Designing 3D skeletons can effectively suppress issues such as dendritic growth, volume change and electrode pulverization during cycling [12–14]. However, with the occurrence of the stripping process, the increasing specific areal surface would lead to more serious electrolyte side reactions [14–18]. In contrast, constructing a robust solid electrolyte interface (SEI) film by interface modification or electrolyte additive strategy is considered to be promising measure to protect Li anode from electrolyte erosion and dendrite growth [8, 19–21]. Usually, despite efficient, it requires the decomposition of additives to form SEI, which is easy to encounter fracture upon repeated Li plating/stripping [22–25]. Therefore, constructing a stable artificial layer directly on lithium can play a vital role in preventing the direct contact between electrode and electrolyte and electrolyte to avoid side reactions and homogenizing the ion flux to inhibit Li dendrites [26, 27].

In order to endow with exceptional ability to uniform Li^+^ flux and accelerate Li^+^ diffusion, it is necessary to consider the component and structure of the interface layer. Substantiated by a large number of literatures, LiF can inhibit dendrite growth due to the low Li^+^ surface diffusion barrier and a high shear modulus (55.1 GPa) [23, 28]. However, its weak adsorption capacity for Li^+^ impedes the homogenize surface Li^+^ flux [29]. It is worth mentioning that carbon-based materials with adjustable electronic structure are often beneficial for heteroatom doping and can be physical barriers to boycott side reactions with electrolyte. The C-F layer prepared by the composite of LiF and carbon-based materials has a great improvement [30–32]. But the interlayer consist of pure inorganic components is brittle, which is not conducive to binding with the Li matrix. Using the perfluoro-organic matter containing a large number of C-F bonds as an interlayer can bring better binding with the Li matrix whereas the strength of the interlayer is limited [33]. For realizing the practical application, to construct organic/inorganic hybrid interlayers with LiF/carbon-based materials/C-F bonds will effectively overcome the above shortcomings.

Herein, we used fluoropolymer grease to engineer organic/inorganic hybrid interlayers (tribofilms), which are derived from the tribochemical reaction between fluoropolymer grease and Li in the rolling process. Multiple crucial advantages can be achieved for constructing organic/inorganic hybrid interlayers (LiF/LiC_6_ inorganic framework hybridized -CF_2_-O-CF_2_- chains) onto the Li metal anode (Li@CFO); (1) the lower surface potential (SP, 42 mV of Li@CFO *vs.* 1.37 V of Li) and excellent lithiophility of the robust interlayers, (2) suppression of lithium dendrite growth, (3) two-dimensional plating of lithium during repeated plating/stripping and (4) the notable desolvation effect to enable fast electrochemical kinetics for Li deposition. All of the above realize the applications with high current densities and high surface capacity. A few microns thickness of the interlayers (~ 2 μm) onto Li@CFO anodes give better electrochemical performance than that of bare Li. The Li@CFO||Li@CFO symmetrical cell shows long cycling stability of up to 5,600 h (more than 2,800 cycles) at 1.0 mA cm^−2^, 1.0 mAh cm^−2^. Surprisingly, it runs more than 540 h (more than 1,350 cycles) even at 18 mA cm^−2^, 3.0 mAh cm^−2^, which is much superior to Li||Li symmetrical cells. What’s more, the LiFePO_4_(LFP)||Li@CFO full cell (the single-side mass loading of LFP ~ 16 mg cm^−2^) exhibits highly stable cycling lifespan with almost 99.9% capacity retention after 450 cycles, whereas the LFP||Li cell failures rapidly. The extremely stable electrochemical behaviors of the Li@CFO anodes can be attributed to the lithiophilic. The presence of -CF_2_-O-CF_2_- into interlayers mainly change the solvation structure composed of Li near the interface, so that the original solvent-separate ion pair (SSIP) and contact ion pair (CIP) mechanisms are converted to aggregates (AGG) mechanism. These characteristics inhibit dendrites growth, induce fast two-dimensional Li plating and stripping, and alleviate the corrosion of the lithium anodes matrix insides with electrolytes. This work paves a new way for scalable productions of high-performance highly stable Li anodes.

## Experimental Section

### Fabrication of Lithium Anodes with C-F-O Interlayer

The Fluoropolymer grease was prepared by modulating and stirring the polytetrafluoroethylene (PTFE, 40 wt%, Aladdin) and PFPE oil (60 wt%, Aladdin) at 45 °C. Different thickness (150, 100, and 50 μm) of Li@CFO was processed by using the grease to coat rollers of the mill (MRX-DG150L, Shenzhen Mingruixiang Automation Equipment Co., Ltd) based on 200 μm Li strips (99.9%, China Energy Lithium Co., Ltd, Tianjin).

### Fabrication of Cathodes

#### LiFePO_4_ Cathode

The cathode slurry comprising LiFePO_4_, super P and polyvinylidene fluoride (PVDF) (Dongguan Large Electronics Co., Ltd. Guangdong, China) at the mass ratio of 94.7%:2.65%:2.65% were fully dispersed in N-methylpyrrolidone (NMP, Aladdin) for half an hour, then coated onto the 14 μm C@Al foil and dried overnight under vacuum at 120 °C. LiFePO_4_ cathode had an active material loading of 10.0 mg cm^−2^ (single side) and the cathode thickness after rolling was 0.085 mm and 16.0 mg cm^−2^ (single side) and the cathode thickness without rolling.

#### S/C Cathode

Sulfur powders (Alfa Aesar) and carbon nanotube (Zhongke era) (mass ratio = 6:4) were ground well with a mortar, then sealed the mixture in an Ar-filled ampoule and placed in a muffle furnace (Hefei Kejing) for 48 h at 300 °C (S/C). The cathode slurry comprising S/C, super P and polyvinylidene fluoride (PVDF) at the mass ratio of 8:1:1 was fully dispersed in N-methylpyrrolidone (NMP, Aladdin) for half an hour, then coated onto the 12 μm Al foil and dried overnight under vacuum at 60 °C. S/C cathode had an active material loading of 4.7 mg cm^−2^ (double sides) and the cathode thickness was 0.290 mm.

## Results and Discussion

### Materials Analysis and Formation Mechanism

**Fig. 1:**
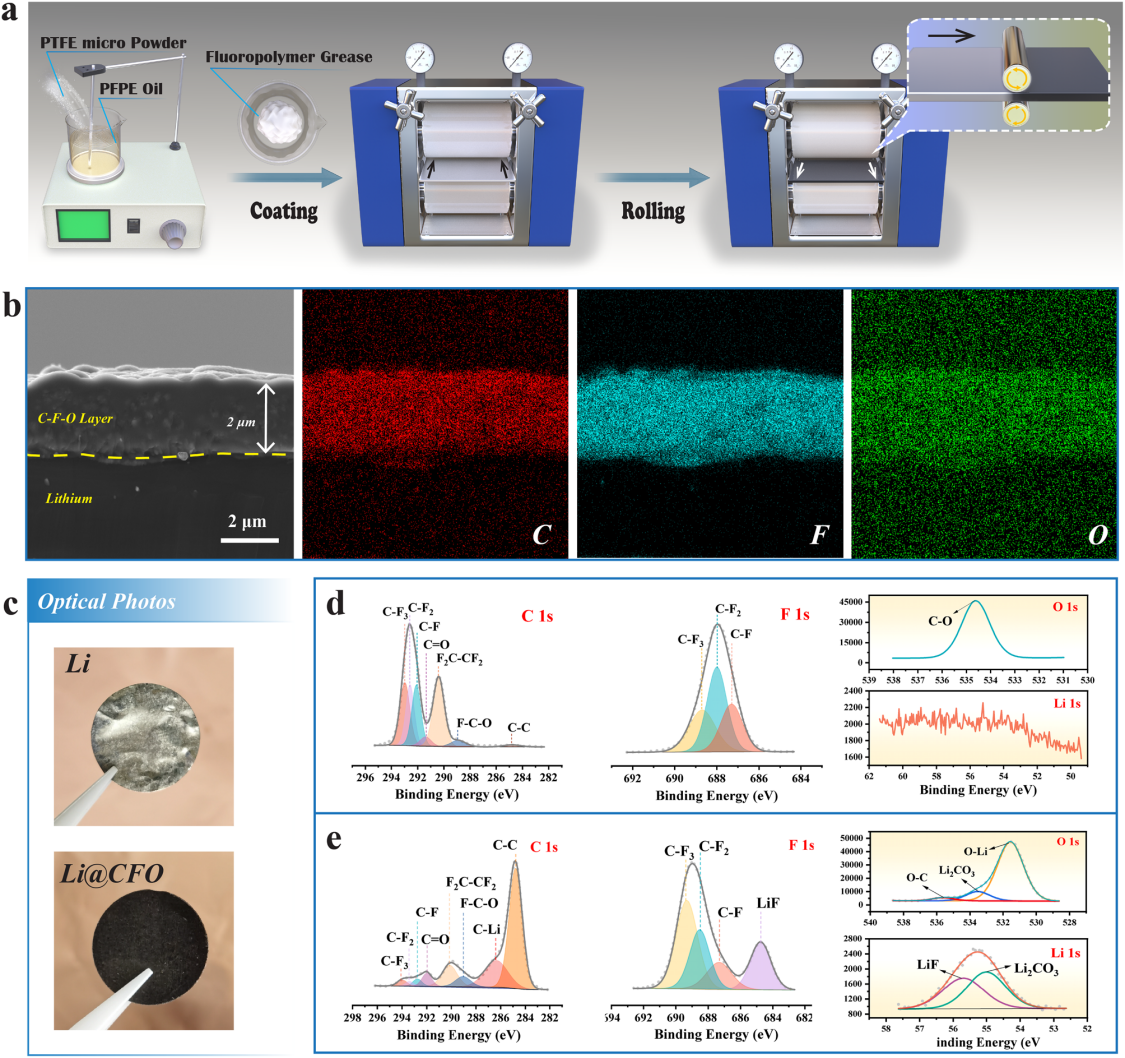
**a** Schematic illustration of the preparation process of Li@CFO anodes. **b** The cross sectional SEM and EDS images of the organic/inorganic hybrid interlayers (~ 2 μm) onto Li@CFO. **c** Optical photos of bare Li and Li@CFO anodes. XPS peak differentiation imitating analysis of C 1*s*, F 1*s*, O 1*s* and Li 1 s onto **d** the interlayers of only coating with the PFPE grease and **e** the organic/inorganic hybrid interlayers of coating then rolling with the PFPE grease

Figure 1a shows the fabrication process of the organic/inorganic hybrid interlayers onto Li@CFO anodes (Fig. S1). The fluoropolymer grease consists of solid phase (PTFE, Fig. S2) and liquid phase (PFPE oil, Fig. S3). Through a simple thinning process after coating the grease onto the rollers, the thickness of the C-F-O interlayers can be controlled at ~ 2 μm approximately shown in Fig. 1b. It indicates that this method is both efficient and reproducible. The prepared Li@CFO anodes appeared obvious black under optical observation (Fig. 1c) while scanning electron microscope (SEM) image and interface roughness images by atomic force microscopy (AFM) tests are shown in Figs. S4-S5. It is worth mentioning that the C-F-O interlayers were formed by reacting with Li metals when loading with high stress. Conversely, the C-F-O interlayers cannot be obtained by coating alone. This was also confirmed in the X-ray photoelectron spectroscopy (XPS) test. Analyzing the chemical structure and electronic information of the Li coating with PFPE grease without rolling, the C 1*s* spectrum can indicate that there are three peaks of 292.9, 292.6 and 292.1 eV at the top layer, corresponding to the three chemical combinations of -C-F_3_, -C-F_2_ and -C-F, respectively (Fig. 1d) [33]. The characteristic peak of PTFE or PFPE at ~ 288.9 eV corresponding to the chemical combination of -F_2_C-CF_2_- merges and the combination of -C–C- at 284.8 eV is not significant. It implies that the PFPE grease does not have a reaction onto the interface. There are no characteristic peak of LiF from the F 1*s* spectrum. Moreover, the XPS signal of Li 1*s* at the interface disappear completely. When the rolling step was performed, the compositions of the interlayers changed significantly. The peak strength of the C–C bond (~ 284.8 eV) is notable increased and the chemical combinations of LiC at 286.2 eV begin to appear in C 1*s* spectrum (Fig. 1e) [34]. This result can speculate about the existence of LiC_6_ components. Analyzing the F 1*s* spectrum, the characteristic peaks of LiF at 684.6 eV merges, which can be assigned to LiF. Combined with the spectra of Li 1*s* and O 1*s*, a small amount of Li_2_CO_3_ would also be present in the reaction products. The appearance of XPS signal of Li 1*s* indicates that the entire interlayer has been fully lithiated after rolling. The original bare lithium surface exists with Li_2_CO_3_ and Li_2_O (Fig. S6) [35].

**Fig. 2:**
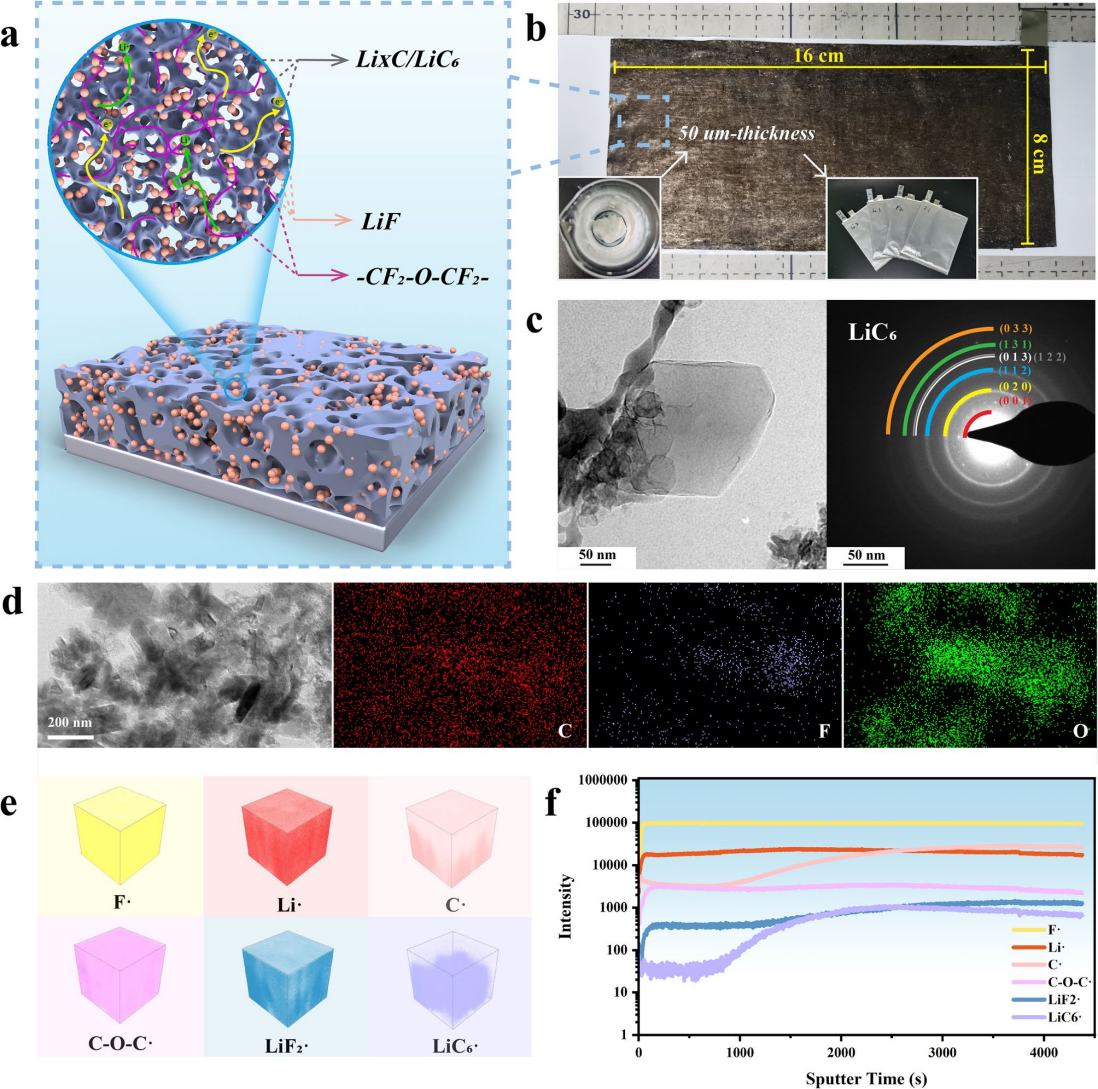
**a** Schematic diagram with the composition and structure of the organic/inorganic hybrid interlayers onto Li@CFO anodes. **b** Optical photograph of the 50-μm Li@CFO anode for the preparation of pouch cells. **c** Morphology and diffraction of the C-F-O interlayers and **d** EDS mapping images under the cryo-TEM. **e** 3D structure views for TOF–SIMS depth sputtering on the surface of Li@CFO. **f** The TOF–SIMS profiles of different atom counts with the depth increasing on Li@CFO

Based on the composition from XPS results, the simplified schematic diagram of the C-F-O interlayer is shown in Fig. 2a. The organic/inorganic hybrid interlayers with LiF/LiC_6_ inorganic framework hybridized -CF_2_-O-CF_2_- chains onto the Li metal anode (Li@CFO) was constructed by tribochemical reaction. These modified anodes for large-scale applications can be easily prepared. An optical photo of a 50 μm-thickness Li@CFO for fabricating pouch cells is shown in Fig. 2b. To further determine the components of the hybrid interface after reacting, cryo-transmission electron microscopy (cryo-TEM) was used to analyze its crystal structure and electronic information. The prepared interlayer was scraped off the surface, then dispersed with 1,2-Dimethoxyethane (DME) solution, and dripped onto the copper net for test. Polycrystalline electron diffraction pattern of LiC_6_ could be observed (Fig. 2c). This also implied that the reaction between PTFE and excess lithium is quite complete. The EDS mapping images (Fig. 2d) are consistent with the previous cross sectional images (Fig. 1b). Moreover, to gain an in-depth understanding of the in situ tribochemical reaction, time-of-flight secondary ion mass spectrometry (TOF–SIMS) test was applied to analyze the components of the longitudinal distribution. It is worth noting that the presence of C–O–C· indicates the original PFPE oil had not reacted in the obtained organic/inorganic hybrid interlayers after rolling (Fig. 2e). In addition to the two obvious components of LiF and LiC_6_, the C–O–C from the PFPE oil was also present in the interlayer. As shown in Fig. 2f, the content of LiC_6_ increases with increasing sputtering depth as well as C. This is mainly because the solid phase component (PTFE) in fluoropolymer grease causes friction reaction on the surface of lithium metal, when grease was impacted by high pressure loads during the rolling process. Thus, PTFE enrich to the interface on Li metal and the following reaction occurs [30]:

$$3\left[ {{\text{CF}}_{2} - {\text{CF}}_{2} } \right]_{n} \left( {{\text{PTFE}},\;{\text{solid phase}}} \right) + 13n\;{\text{Li}}\xrightarrow{{^{{{\text{Friction by Rolling}}}} }}n\;{\text{LiC}}_{6} + 12n\;{\text{LiF}}$$

The main inorganic components in the C-F-O interlayer are obtained by this reaction while organic component gained from original fluoropolymer grease. The PFPE oil provides -CF_2_-O-CF_2_- chains to hybridize the LiF/LiC_6_ inorganic framework.

### Lithium Anodes Performance Testing in Symmetrical Cells

**Fig. 3:**
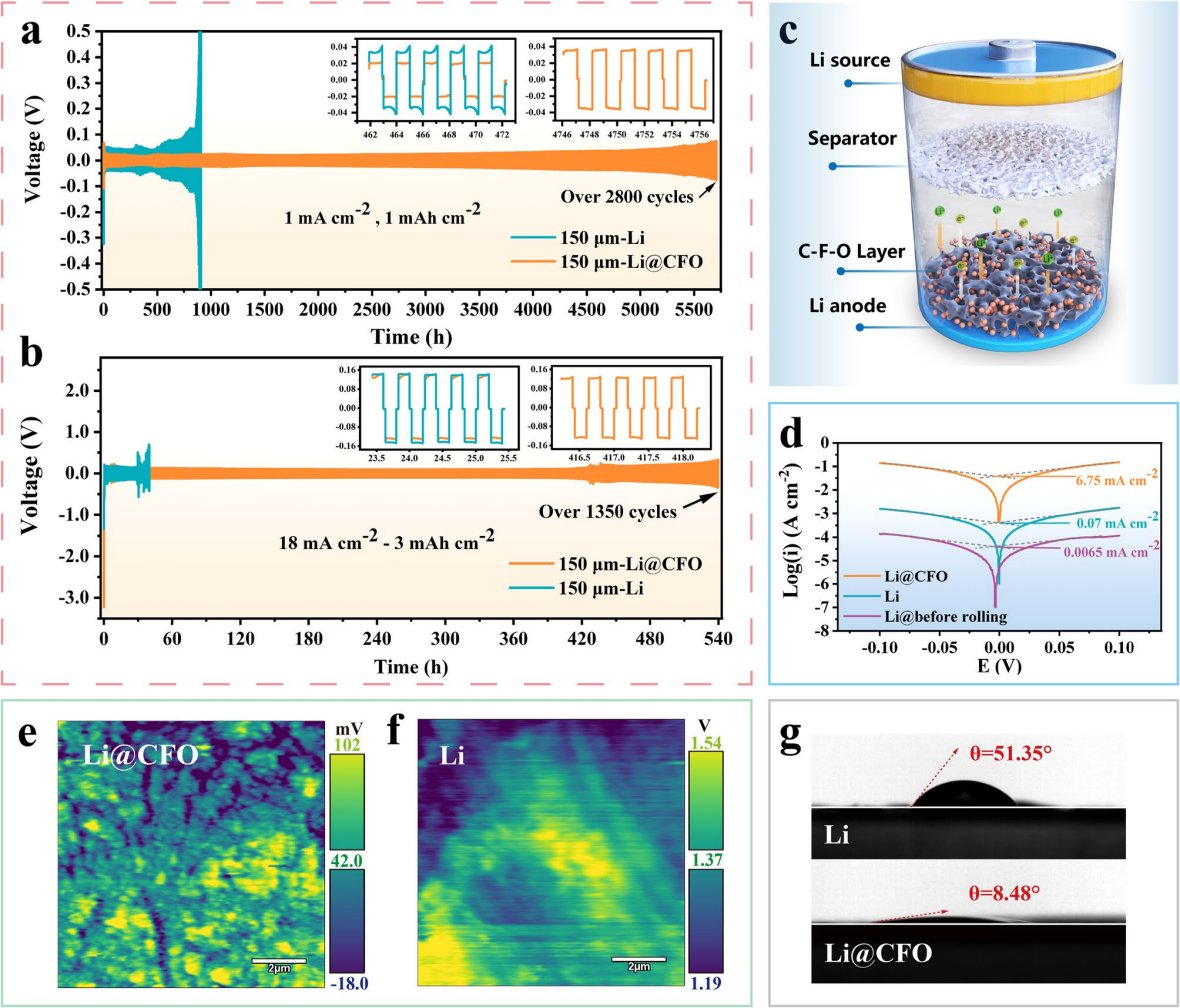
**a** Galvanostatic plating/stripping test of symmetric cells with Li@CFO (150-μm-thickness) and bare Li (150-μm-thickness) electrodes at 1.0 mA cm^−2^ and 1.0 mA h cm^−2^ using LS009 electrolyte. **b** Galvanostatic plating/stripping test of symmetric cells with Li@CFO (150-μm-thickness) and bare Li (150-μm-thickness) electrodes at 18.0 mA cm^−2^ and 3.0 mAh cm^−2^ in the LS009 electrolyte. **c** Schematic diagram of the electrochemical mechanism on the Li@CFO anodes. **d** Tafel curves of bare Li and Li@CFO anodes under a constant voltage of 100 mV. Surface potential mapping of **e** Li@CFO and **f** bare Li under KPFM measurement. **g** Contact angle tests of LS009 electrolyte drop with bare Li and Li@CFO

The C-F-O interlayer has a significantly positive act on the electrochemical performance. At 1.0 mA cm^−2^ and 1.0 mAh cm^−2^, Li@CFO||Li@CFO symmetrical cell achieves a long lifespan up to 2,800 cycles (over 5,600 h) using 1 M LiTFSI in DME:DOL = 1:1 Vol% with 2% LiNO_3_ (LS009 electrolyte) shown in Fig. 3a. Even at high current density of 18.0 mA cm^−2^ and practical-used areal capacity of 3.0 mAh cm^−2^, more than 1,350 cycles (over 500 h) can be achieved at Li@CFO||Li@CFO cell (Fig. 3b). In contrast, the polarization voltage of Li||Li symmetrical cell significantly increases at less than 400 cycles (~ 800 h) over 150 mV at 1.0 mA cm^−2^ and 1.0 mAh cm^−2^ while increases at less than 75 cycles (~ 30 h) over 500 mV at 18.0 mA cm^−2^ and 3.0 mAh cm^−2^. At the test conditions of 2.0 mA cm^−2^ and 1.0 mAh cm^−2^, Li@CFO can also sustain a lifespan of more than 780 h, while bare lithium fails after about 240 h (Fig. S7). The 150 μm thickness of electrodes were used in the above cyclic charge–discharge tests. To quantify the lithiophility of Li@CFO, the overpotential of first cycle in galvanostatic plating/stripping tests were analyzed. At low current density of 1.0 mA cm^−2^ and low areal capacity of 1.0 mAh cm^−2^, the overpotential of Li@CFO is only at 8 mV while bare Li even reach 120 mV. As shown in Fig. S8 (Supporting Information), SEM and EDS images indicate the 2D-non-dendrite growth was realized under the induction of the C-F-O interlayer. Throughout the electro cycles, the C-F-O interlayers protect the lithium metal matrix, acts as a depositary layer and transports lithium ions shown in Fig. 3c. In Fig. 3d, the Li@CFO exhibits a higher exchange current density of 6.75 mA cm^−2^ in relative to that of bare Li (0.07 mA cm^−2^), indicating that the electrochemical kinetics in Li@CFO are enhanced with constructed LiF/LiC_6_ framework hybridized -CF_2_-O-CF_2_- chains of C-F-O interlayers. What’s more, the Li anode by only coating without rolling exhibits a quite lower exchange current density of 0.0065 mA cm^−2^, which was verified the occurrence of the friction reaction from the other side. To further verification of the characteristics of electron conductivity on C-F-O interlayer, Kelvin probe force microscopy (KPFM) tests were used to explore the local surface potential (SP) on different anodes (Fig. 3e-f). It can be observed that the local interface potential of the Li@CFO decreases a lot compared with bare Li. From the SP mapping, the areal average potential of Li@CFO is ~ 42.0 mV, while bare Li enriched Li_2_O and Li_2_CO_3_ on the surface is ~ 1.37 V. The lower SP facilitates the faster deposition of lithium ions on the interlayer from the solvent. Under the action of the characteristics of the C-F-O interlayer, the dense island-like two-dimensional growth at current density of 18.0 mA cm^−2^ after 30 cycles was realized (Fig. S9). While bare Li underwent severe dendrite growth, eventually leading to rapid failure (Fig. S10).

### Mechanism of Electrochemical Behaviors

**Fig. 4:**
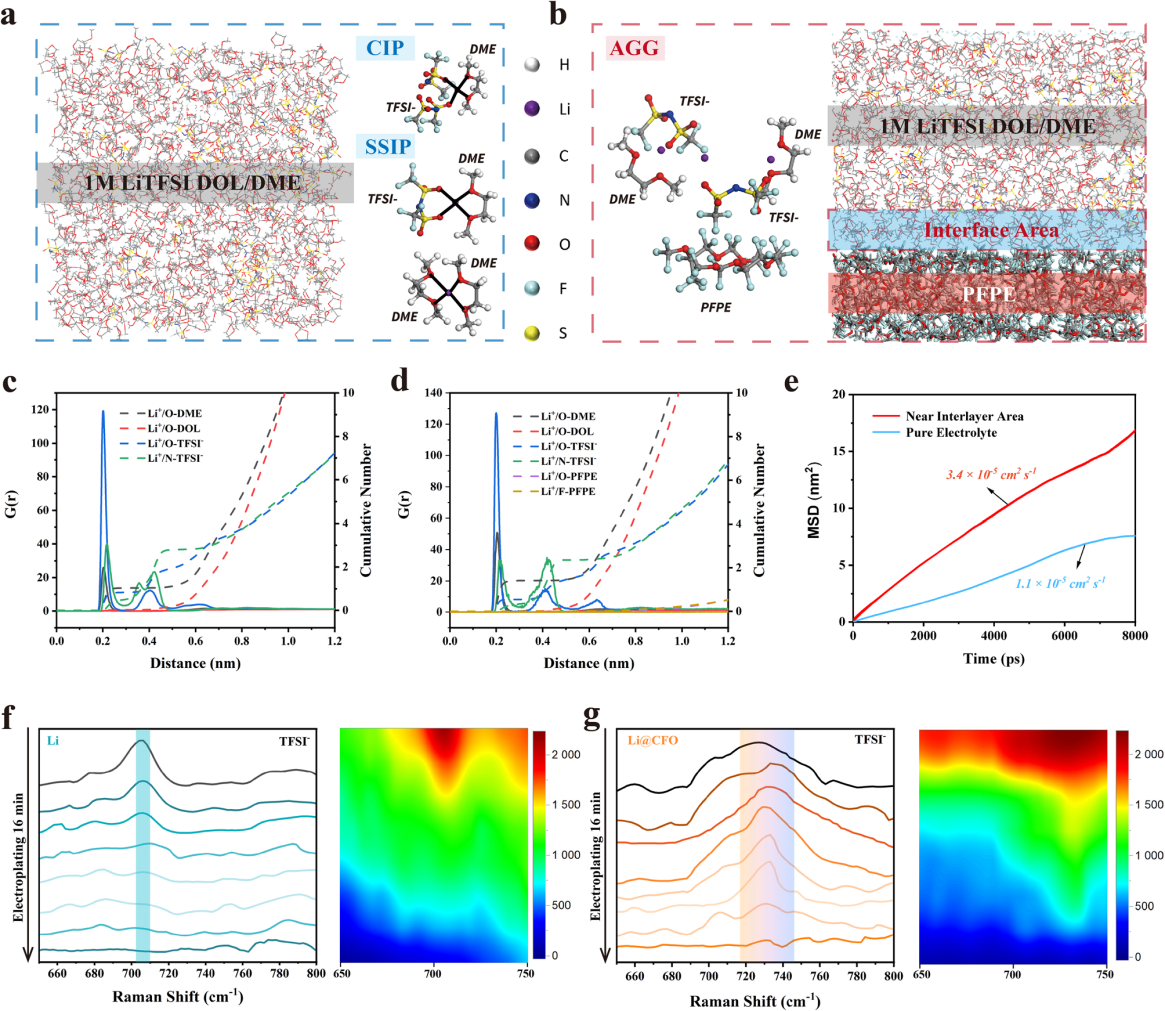
**a** Snapshots of the 1.0 M LiTFSI in DME:DOL = 1:1 Vol% solvation environment. **b** Snapshots of the solution environment in the C-F-O interlayer area. **c** The radial distribution function g(r) (solid lines) and cumulative number (dotted lines) of the O atoms on DOL/DME molecules around Li-ions in 1 M LiTFSI/DOL-DME electrolyte. **d** The radial distribution function g(r) (solid lines) and cumulative number (dotted lines) of the O atoms on DOL/DME molecules around Li-ions in the C-F-O interlayer area. **e** Li^+^ diffusion coefficient under system *a* and system *b*. In situ Raman spectra of electrolyte near anode–electrolyte interface during Li (**f**) and Li@CFO plating (**g**) with 1.0 M LiTFSI in DME:DOL = 1:1 Vol% at a plating current density of 3 mA cm.^−2^

In order to further explore the local desolvation dynamics environment of Li^+^ near the C-F-O interlayer, the interaction between the C-F-O interlayer and the local electrolyte environment near the interlayer needs to be revealed. Using LS009 electrolyte, the contact angle with Li@CFO is 8.48°, which is much smaller than that with bare Li (~ 51.35°). The smaller contact angle confirms the better wettability shown in Fig. 3g due to the rougher interface (Fig. S5). The roughened interface reduces the actual current density, which is beneficial for inhibiting dendrite growth at high current densities [12, 36, 37]. The interaction between the interface and electrolyte can be explored using molecular dynamics simulation. As shown in Fig. 4a, b, it exhibits that organic ingredient of Li@CFO anode effectively reduces the coordination number of lithium ions. In pure electrolyte system, a typical solvated configuration under an equilibrium system is characterized by 4 coordination. The main manifestations are contact ion pair (CIP) and solvent-separate ion pair (SSIP) and CIP is the main configuration (Fig. 4a) [38]. Adding an organic interlayer, a typical solvated configuration at the interface under the equilibrium system is 3 coordination and below. The main manifestation at the interface is aggregates (AGG) as shown in Fig. 4b. It means that the interface after fluoropolymer grease reaction can effectively achieve lithium-ion desolvation. It can be known from the radial distribution function g(r) (the dotted lines shown in Fig. 4c-d) that PFPE is a neutral organic molecule and does not participate in the solvation structure of Li, but it changes mainly the solvation structure composed of Li at the interface with DME and TFSI-, so that the original SSIP and CIP states converse to AGG state. Analyzing the integration of the radial function, it is seen that the introduction of PFPE causes the coordination number of Li–O to be converted from 4 to 3 or less, which means that the kinetics characteristics of Li will be enhanced and have a great help in enabling fast stripping/plating of lithium ions at the solid/liquid interface [39]. To determine the self-diffusion coefficient *D*_*Li*_ of particles of type Li, can use the Einstein relation and the mean square displacement (MSD) and *D*_*Li*_ are calculated by the program GROMACS using the following formula [40]:

$$\mathop {{\text{lim}}}\limits_{{t \to \infty }} \left\langle {\left\| {r_{i} \left( t \right) - r_{i} \left( 0 \right)} \right\|} \right\rangle _{{i \in {\text{Li}}}}^{2} = 6{\text{D}}_{{{\text{Li}}}}$$

By calculating the relationship between mean square displacement MSD and time t of lithium ions in pure electrolyte in Fig. 4a and electrolyte with interlayer area in Fig. 4b (shown in Fig. 4e), the diffusion coefficient of lithium ions in the two systems can be obtained. It is found that the diffusion rate of lithium ions (~ 3.4 × 10^–5^ cm^2^ s^−1^) in the electrolyte with interlayer area in Fig. 4b is about 3 times that of the system of pure electrolyte in Fig. 4a (~ 1.1 × 10^–5^ cm^2^ s^−1^), and the higher diffusion coefficient will increase the transmission rate of lithium ions. To investigate the Li^+^ solvation structure near the Li or Li@CFO interface, Raman spectral tests were conducted using 1.0 M LiTFSI in DME:DOL = 1:1 Vol% electrolyte. Figure 4f-g shows Raman spectra results, where the S–N–S bending peak of the TFSI^−^ at 731.20 cm^−1^ (near the Li@ZDDP interface) undergoes redshift when dissolved in DOL/DME solvents. While the S–N-S peak bending peak of the TFSI^−^ at 708.60 cm^−1^ near the bare Li interface undergoes redshifted compared to the Li@CFO. With the electrodeposition process, the peak strength gradually disappears. This implies a high degree of solvation near the bare lithium interface during the plating. On the contrary, the S–N-S peak near the Li@CFO interface underwent blueshift with the progress of electroplating. This indicates that there is much stronger cation–anion interaction near the Li@CFO interface and the desolvation effect is significant herein [41]. Therefore, the Li@CFO anodes exhibit better performance at larger current densities [42].

**Fig. 5:**
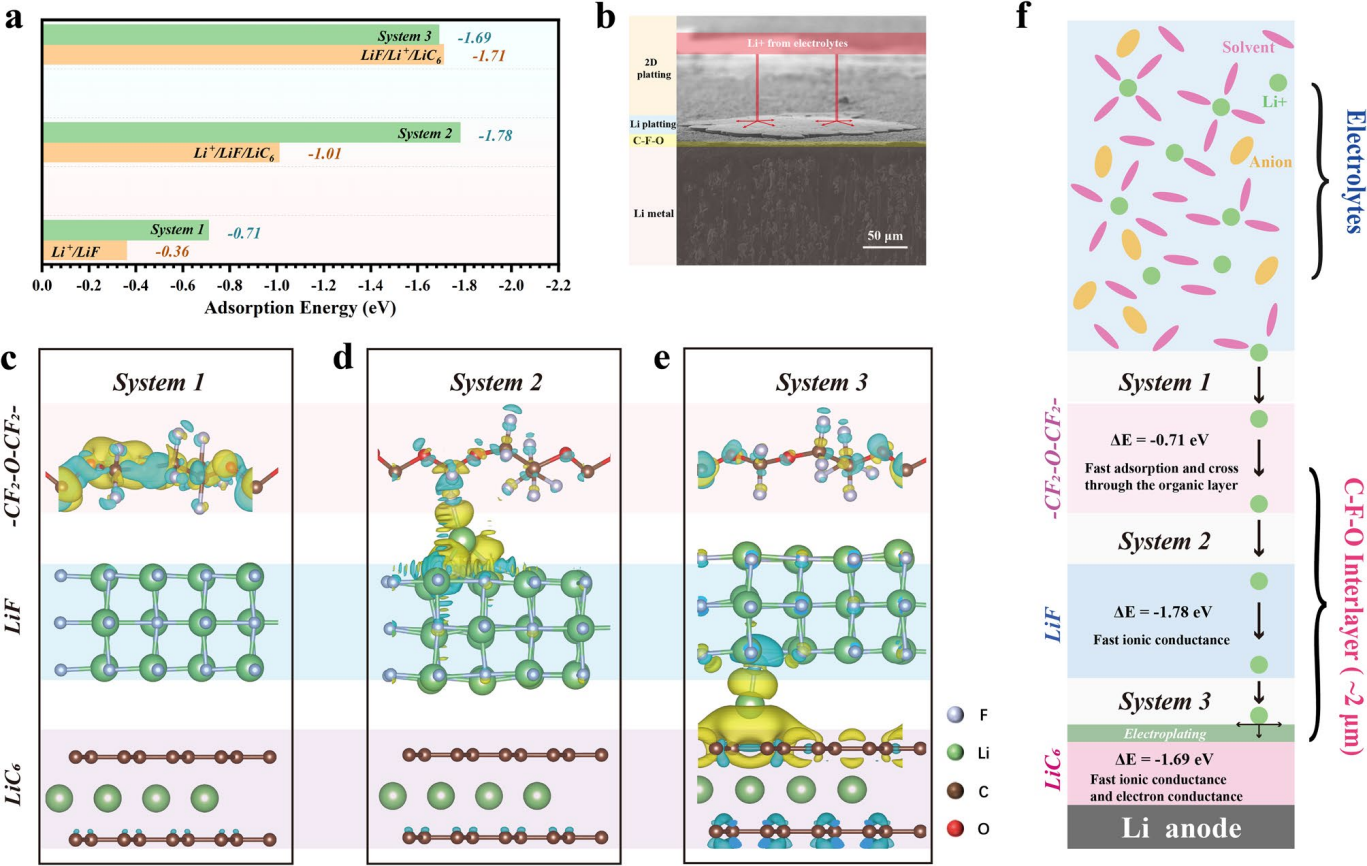
**a** Adsorption energies of Li^+^ in different systems. **b** The cross sectional SEM with deposition schematic image of the Li@CFO after cycling at 1.0 mA cm^−2^ and 3.0 mA h cm^−2^. **C–e** Li^+^ diffusion path through the C-F-O interlayer. *System 1* is Charge density difference plot of a Li^+^ on the -CF_2_-O-CF_2_- of PFPE in the C-F-O interlayer. *System 2* is a Li^+^ on the LiF (001) facet in the C-F-O interlayer. *System 3* is a Li^+^ on the LiC_6_ in the C-F-O interlayer. **f** Schematic diagram of the electrochemical mechanism of Li^+^ diffusion and plating path on the C-F-O interlayer

To reveal the mechanism of the C-F-O interlayer on fast dynamics process and 2D deposition behaviors, density functional theory (DFT) was conducted to analyze the interaction relationships on Li^+^ with LiF, LiF/LiC_6_, or C-F-O interlayer. The adsorption energy of Li^+^ and charge density distribution of LiF, LiF/LiC_6_, or C-F-O interlayer are summarized in Fig. 5a. Figure 5c-e describes the situations of Li^+^ through the C-F-O interlayer. The process is shown in Fig. 5f. Lithium ions in the electrolytes interact with the organic layer firstly. In this case, the adsorption energy of Li^+^ on -CF_2_-O-CF_2_- is −0.71 eV. The organic C−F bonds have a stronger Li^+^ adsorption than LiF (−0.36 eV). It also greatly reduced the adsorption energy in *system 2* (Fig. 5d) of Li^+^/LiF/LiC_6_ (Fig. S11), which indicates PFPE further improves the lithophilicity of LiF. While the adsorption energy of LiF/Li^+^/LiC_6_ (Fig. S11) shows not significant differences with *system 3*. It shows that the transport of Li^+^ between LiF and LiC_6_ is not affected by the organic layer. Overall, the system by hybridizing -CF_2_-O-CF_2_- chains can enhance the adsorption of Li^+^ greatly, improving the lithophilicity of the entire interlayer [43]. Lower adsorption energy is able to preuniform Lewis acid Li^+^ flux and beneficial to homogeneous Li^+^ flux for further deposition [8, 30, 44]. The great lithiophilicity enables a uniform distribution of Li ion flows near the interface, ultimately enabling 2D deposition (Fig. 5b) [45, 46]. Meanwhile, the extremely fast ionic conductivity and extremely strong ion adsorption inhibits dendrite growth at high current densities [47].

### Lithium Anodes Performance Testing in Full Cells

**Fig. 6:**
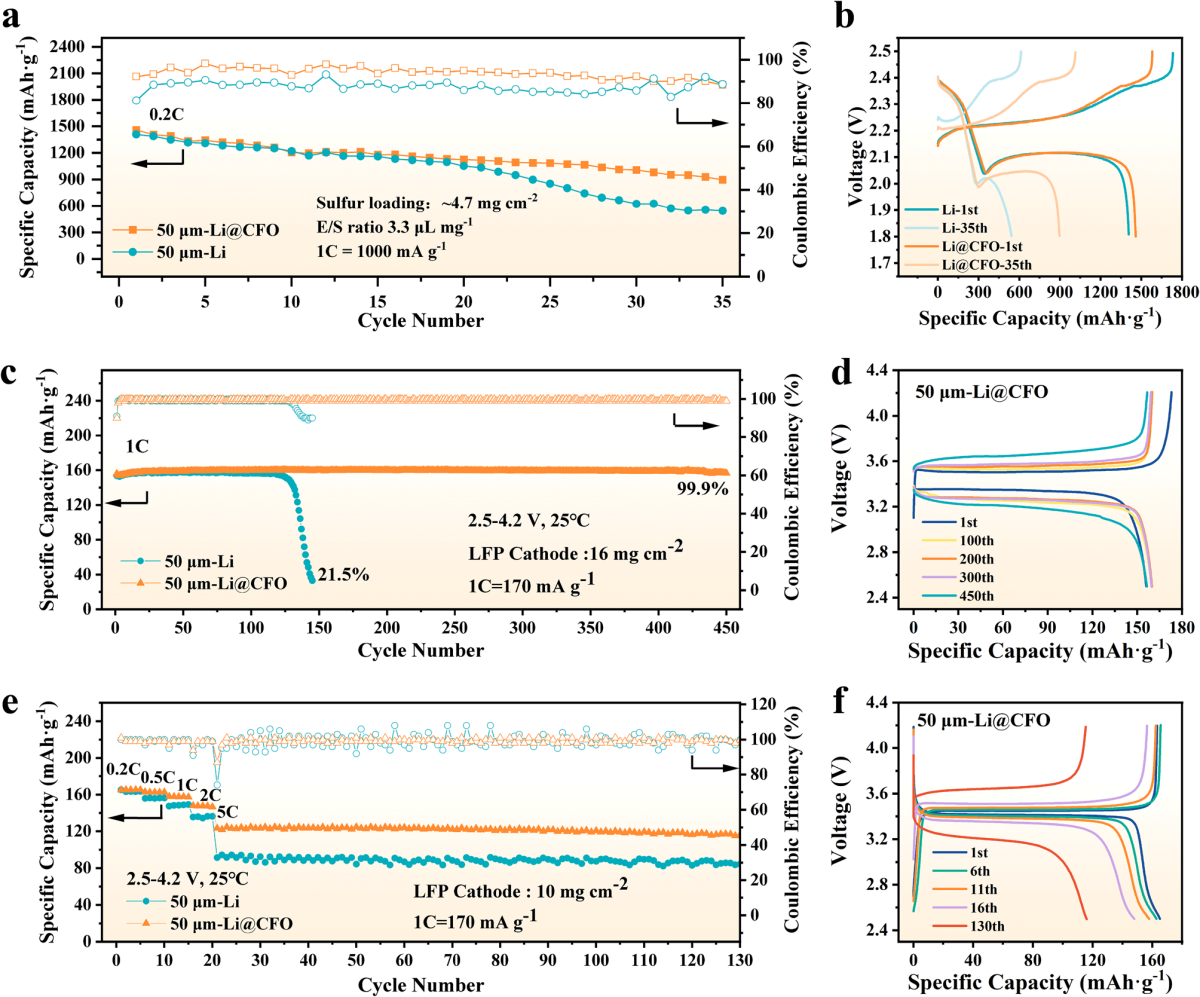
**a** Cycling performance of the pouch cell with S||Li@CFO (50 μm) using 1.0 M LiTFSI in DME:DOL = 1:1 Vol% with 2.0% LiNO_3_ electrolyte. **b** Galvanostatic charge/discharge profiles at different cycles using pouch cell with 50 μm anode. **c** Cycling performance of the LFP||Li and LFP||Li@CFO full cells at 1C rate in 1.0 M LiPF6 in EC:EMC:FEC = 3:7:1 Vol% electrolyte. **d** The charge–discharge profiles of LFP||Li@CFO at 1C rate after cycling. **e** Cycling performance of LFP||Li and LFP||Li@CFO full cell at different rates and take charging/discharging at 5C rate. **f** The corresponding voltage profiles of LFP||Li@CFO cells

To investigate the application prospect of the prepared Li@CFO anodes in the practically rigorous conditions (such as high mass loading cathode and lean electrolyte/sulfur ratio) of commercial cells, S||Li@CFO and LFP||Li@CFO were assembled, respectively. The performance of lithium–sulfur pouch cell was verified in Fig. 6a. The electrolyte–sulfur (E/S) ratio was 3.3 μL mg^−1^ to evaluate the performance of the 50 μm-thickness Li@CFO anodes. Due to the organic–inorganic hybrid interlayer onto anodes, reducing the corrosion of polysulfides with Li metal from the electrolytes, the capacity of the pouch cell would stay highly stable than that of bare Li [41, 48]. Under the lean E/S ratio state, the discharging specific capacity of S||Li@CFO battery can still maintain ~ 900 mAh g^−1^ (vs. 540 mAh g^−1^ of bare Li). The lean electrolyte and free Li were continuously reacted resulting the dendrite growth and sulfur corrosion on Li metal, which induced rapid failure in the S||Li cell. This trend is evident from the specific capacity–voltage curves shown in Fig. 6b. Routine 1C cycle test was performed with 16 mg cm^−2^ mass loading of LiFePO_4_ (LFP) cathode, where the current density reach ~ 2.9 mA cm^−2^ in LFP||Li@CFO achieving in excess of 450 cycles with a capacity retention rate of up to 99.9%. On the contrary, the bare Li anode occurred severe short circuit due to the dendrite growth, causing the discharging specific capacity declined rapidly as shown in Fig. 6c. Figure 6d exhibits that the discharge specific capacity almost did not decline at 1C rate while that of Li declined fast (Fig. S12). Discharge rate tested from 0.2C, 0.5C, 1C, 2C to 5C, the discharge specific capacity of Li@CFO anode always stays high and stable as compared with that of 50-μm-thickness bare Li anode (Fig. S13). Furthermore, even under a rigorous condition such as 10 mg cm^−2^ mass loading of LFP cathode, the LFP||Li@CFO can still discharge a high specific capacity of ~ 125 mAh g^−1^ at 5C rate while the LFP||Li can only discharge a specific capacity of ~ 90 mAh g^−1^ shown in Fig. 6e, where the current density reach ~ 7.6 mA cm^−2^, indicating the extraordinary rate performance of the Li@CFO anode. Figure 6f confirms that the discharge specific capacity does not decline much at different rates. Through the distribution of the floating histogram of Coulombic efficiency at 5C rate, it can be seen that the modified Li@CFO anodes show a higher stable charge–discharge behaviors (Fig. S14).

## Conclusions

In summary, a stable thin Li anode with an organic/inorganic hybrid interlayer was developed by the in situ friction reaction between fluoropolymer grease and Li strips during rolling. Designing in situ friction reaction follows some principles. Firstly, the reaction should be controllable. In general, friction reactions on the surface of alkali metals are more dangerous [49]. Try to avoid using pure solid phases with high chemical activity. Secondly, there is almost no reaction between the reactants and the lithium metal when directly contacting. In this case, the reaction based on friction can obtain the micro-nano scale of interlayer. Thirdly, reaction product components need to be designed. The reaction product components might contain the SEI components as literature reported. Ensure the electrochemical activity of the interlayer. The constructed organic -CF_2_-O-CF_2_- chains onto Li@CFO anodes can effectively build local desolvation environment to realize the fast reversible Li stripping/plating processes. Moreover, the two-dimensional plating behaviors can alleviate the volume changes and the Li dendrite growth. The as-prepared lithium anode with C-F-O interlayer exhibits a prolonged cycle lifespan and high-rate cycle stability, which is in excess of 2,800 cycles (over 5,600 h) at 1.0 mA cm^−2^&1.0 mAh cm^−2^ and more than 1,350 cycles (over 500 h) even at 18.0 mA cm^−2^ and 3.0 mAh cm^−2^. What’s more, the LiFePO_4_||Li@CFO full cell last over 450 cycles at 1C with a high-capacity retention of 99.9% and stay 110 cycles ~ 120  mAh g^−1^ at 5C. This work provides a facile, green and scalable approach concerning friction design for producing stable lithium metal anodes.

### Supplementary Information

Below is the link to the electronic supplementary material.

Supplementary file1 (PDF 1868 KB)
