# Supplementary material for: In Situ Formed Tribofilms as Efficient Organic/Inorganic Hybrid Interlayers for Stabilizing Lithium Metal Anodes
Source: Nanomicro Lett. 2023 Oct 24;15:235. doi: 10.1007/s40820-023-01210-6 (PMC10597943; doi:10.1007/s40820-023-01210-6)
Supplement: Supplementary file 1 — Supplementary file1 (PDF 1868 KB) [file 40820_2023_1210_MOESM1_ESM.pdf]

Supporting Information for

In Situ Formed Tribofilms as Efficient Organic/Inorganic Hybrid Interlayers for Stablizing Lithium Metal Anodes

Shaozhen Huang[1], Kecheng Long[1], Yuejiao Chen[1], Tuoya Naren[1], Piao Qing[1], Xiaobo Ji[1, 2], Weifeng Wei [1], Zhibin Wu[1, *], Libao Chen[1, *]

[1] State Key Laboratory of Powder Metallurgy, Central South University, Changsha, 410083, P. R. China

[2] College of Chemistry and Chemical Engineering, Central South University. Changsha, Hunan 410083, P. R. China

*Corresponding authors. E-mail: zhibinwu@csu.edu.cn (Z. Wu); lbchen@csu.edu.cn (L. Chen)

S1 Methods

S1.1 Materials Characterization

The thickness of the Li anode and surface morphologies of Li electrodes were characterized using thermal field emission SEM (MIRA4 LMH, TESCAN, Czech) operated at 5.0 kV. The XPS measurements were performed by Thermo Scientific K-Alpha$^+$ setup with a monochromatic Al Ka X-ray source and an Ar^+ sputtering gun (Thermo Fisher) from Shiyanjia Lab (www.shiyanjia.com). The sample of cryo-TEM (Spherical aberration correction field emission transmission electron microscope, Titan G2 60-300, FEI) was made by scrapping off the surface layer of Li@CFO and using dimethyl carbonate (DMC) to disperse on copper mesh. Composition of surface was characterized by time-of-flight secondary ion mass spectrometry (TOF-SIMS, Gmhb 5, Münster, Germany) with an Ar-ion beam. The sputtering speed was about 1.0 nm s^{-1}. For bare lithium and Li@CFO, we used atomic force microscopy (AFM, Aist-NT) and Kelvin probe force microscopy (KPFM, Cypher S, Asylum Research, Oxford Instruments) to characterize the surface potential and roughness.

S1.2 In situ Raman Characterization

Raman spectra tests were conducted with a Horiba Jobin Y von HR Evolution Raman spectrometer under a Raman laser wavenumber of 532 nm from a neodymium-doped yttrium aluminum garnet (Nd:YAG) laser operating at 120 mW. The Li deposition in Raman spectroscopic cell (K007, Tianjin Aida Hengsheng Technology Development Co., Ltd) with 1.0 M LiTFSI in DME:DOL=1:1 Vol% was performed at 3 mA cm^{-2} lasting 16 min which implies a theoretical deposition thickness of ~4 μm, while the laser spot has a diameter of ~1.5 μm. For the *in situ* Raman test, the distance between the laser spot and the substrate was set as 5.5 μm.

S1.3 Electrochemical Measurements

The discharge/charge testing were conducted on a Neware Battery system. All electrochemical tests were performed in a temperature-controlled room at 25℃. All batteries employed Celgard®2400 as the separator. *Coin cells tests.* CR2016 coin cells were assembled in an Ar-filled glovebox (O_2 and H_2O < 0.5 ppm). Symmetric coin cells (fresh lithium on each side or Li@CFO foil on each side) were assembled with 1.0M LiTFSI in DME:DOL=1:1 Vol% with 2.0% $LiNO_3$ (LS009, Dodo Chem) as the electrolyte. To standardize the testing, 75 μL of electrolyte was used in each coin cell. Typically, we used a protocol of 1 h of stripping followed by 1 h of plating with a current density of 1.0 mA cm^{-2} to achieve an areal capacity of 1.0 mA h cm^{-2}. The current density for the Li metal plating/stripping was set to 1.0 or 18.0 mA cm^{-2}. Besides, LFP||Li@CFO (or Li) full cells were assembled with 1.0M $LiPF_6$ in EC:EMC:FEC=3:7:1 Vol% (LB515, Dodo Chem) as the electrolyte, which were tested within the voltage range of 2.5-4.2 V. As for cycling tests, the LFP full cells were activated at 0.1C for 2 cycles firstly, then underwent the charge-discharge test at 1C (1C = 170 mA h g^{-1}). As for rate performance tests, the LFP full cells underwent the galvanostatic charge-discharge test in turn of 0.2C, 0.5C, 1C, 2C and 5C, then cycling at 5C (1C = 170 mA h g^{-1}). *Pouch cells tests.* The S||Li@CFO (or Li) pouch cells were assembled in dry rooms with dew points below – 40 °C. The size of the Li anode was 50 mm × 80 mm while the size of the S cathode was 47 mm × 77 mm. Each electrode had the pole lug at the size of 8 mm × 12 mm. The width of the polypropylene separators was 83 mm. In the layer-by-layer manufacturing process, the S||Li@ZDDP (or Li) pouch cells used three cathodes and four anodes while lithium sulfur pouch cells used two cathodes (30 mm × 70 mm) and three anodes (33 mm × 73 mm). Aluminum electrode lugs were welded to the cathodes by an ultrasonic welder while nickel electrode lugs were welded to the anodes by mechanical pressing. S||Li@CFO (or Li) pouch cells were tested over the voltage range of 1.8-2.5 V. Each pouch cell was tested at 0.2C (1C = 1000 mAh g^{-1}). The electrolyte/sulfur (E/S) ratio was 3.3 μL mg^{-1}.

S1.4 Computational Methods

MD simulation was performed using Gromacs (2019.5 version, http://www.gromacs.org). For pure electrolyte system, 300 DME, 420 DOL, 60 Li^+ and 60 $TFSI^-$ were added to 5×5×5 nm simulation box. For system of adding an organic interlayer,150 PFPE were added to 5×5×1.5 nm simulation box as the bottom layer and 250 DME, 350 DOL, 50 Li^+ and 50 $TFSI^-$ were added to 5×5×3.5 nm top layer. The OPLS-AA force field with the charges refitted using RESP approach were adopted. For each system, the simulation started with a 2 ns NPT at 500 K and was followed by a 3 ns NPT at 330 K to make sure for the full dissolution of the electrolyte. Then, the systems were equilibrated at 298 K in the NPT ensemble for 5 ns and in the NVT ensemble for 10 ns. The last 5 ns trajectory is used to obtain the structure and the Radial distribution function (RDF) for the interface selected the regions that more than 1nm away from the bottom layer. *Density functional theory (DFT)* calculations were

performed using the Vienna Ab Initio Package (VASP) with the generalized gradient approximation Perdew-Burke-Ernzerhof (GGA-PBE) functional. Projected augmented wave (PAW) potentials were chosen to describe the ionic cores, and valence electrons were considered using the plane wave basis set with a kinetic energy cutoff of 500 eV. The electronic energy was considered self-consistent when the energy change was smaller than 10−4 eV. The geometry optimization was considered convergent when the residual forces were less than 10-2 eV Å^{-1}.

Supplementary Figures

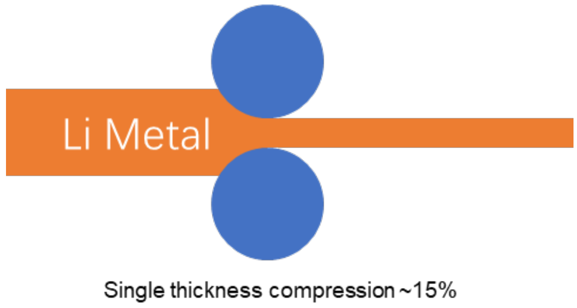

Fig. S1 Schematic diagram of the rolling process for preparing the Li@CFO

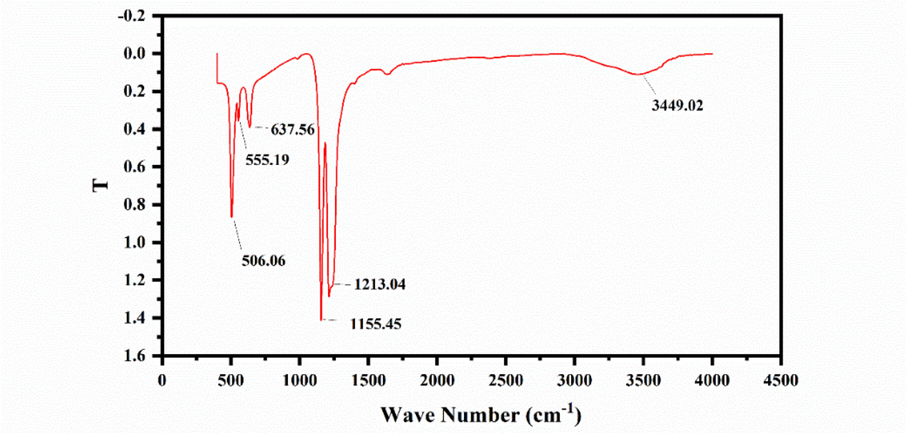

Fig. S2 Image of infrared spectroscopy of pure PTFE

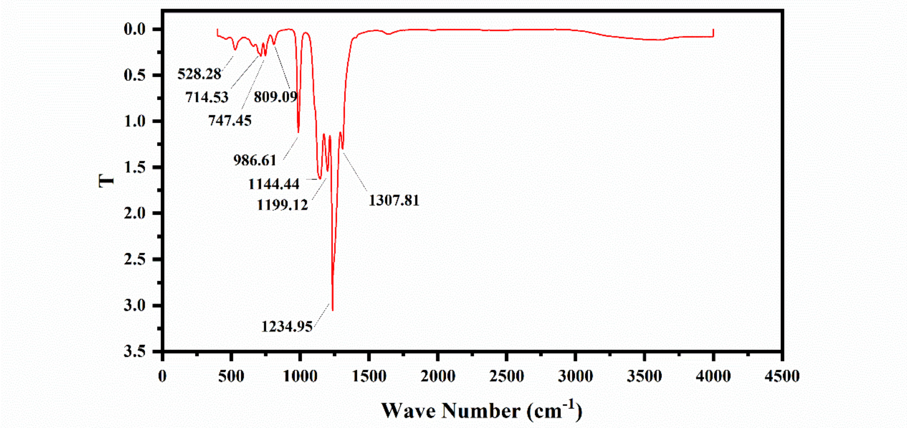

Fig. S3 Image of infrared spectroscopy of pure PFPE

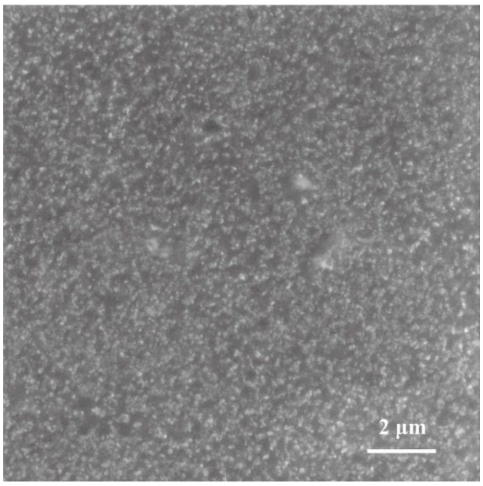

Fig. S4 SEM image of the surface on prepared Li@CFO

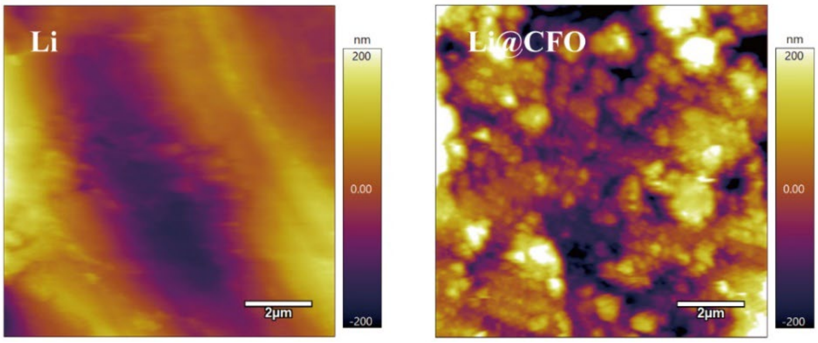

Fig. S5 Interface roughness of Li and Li@CFO by AFM tests

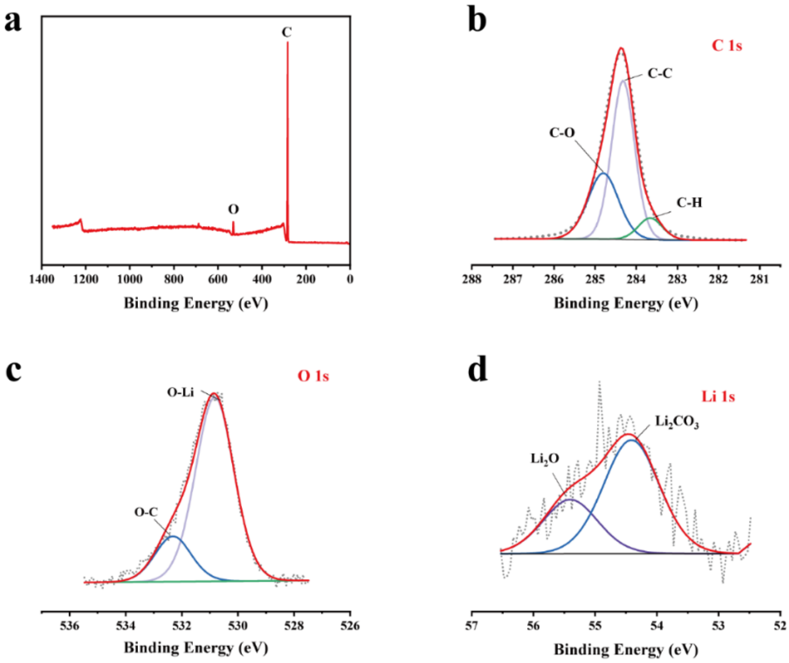

Fig. S6 XPS spectra of bare Li electrode for the initial. (**a**) Survey, (**b**) C 1s spectra, (**c**) O 1s spectra, (**d**) Li 1s spectra

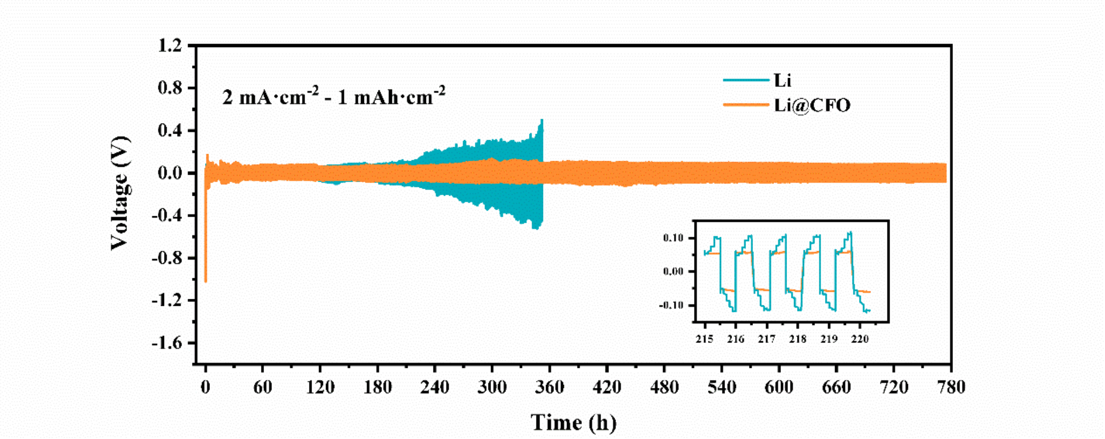

Fig. S7 The voltage-time profiles of symmetrical cells with Li and Li@CFO at 2.0 mA cm^{-2} and 1.0 mA h cm^{-2}

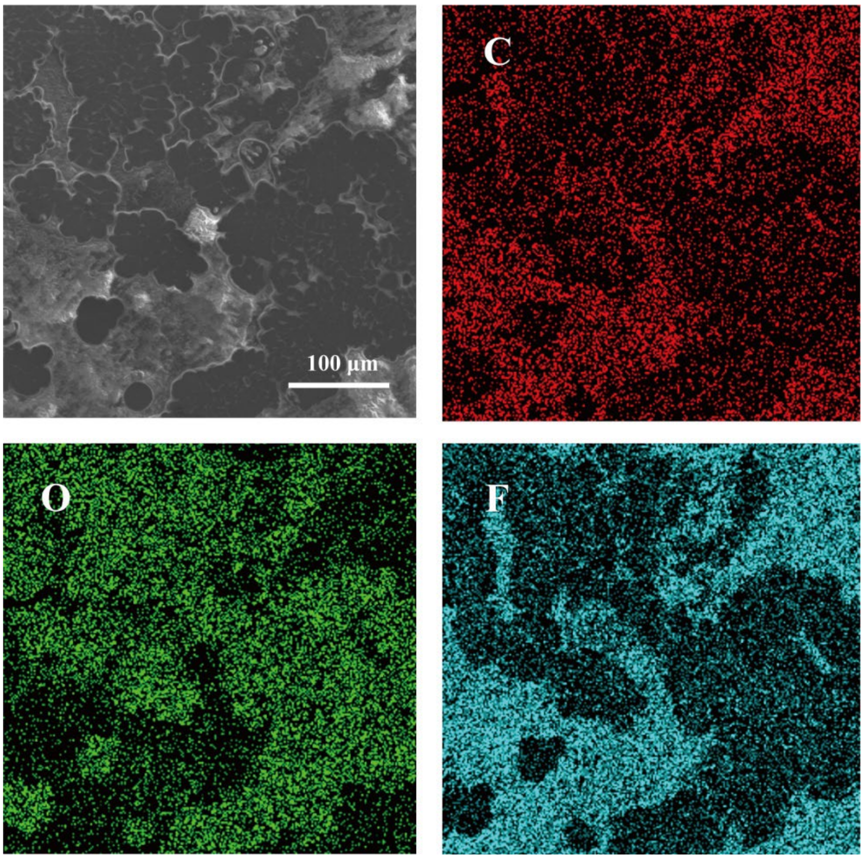

Fig. S8 EDS images of Li@CFO plating after 1 cycle at at 1.0 mA cm^{-2} and 1.0 mA h cm^{-2}

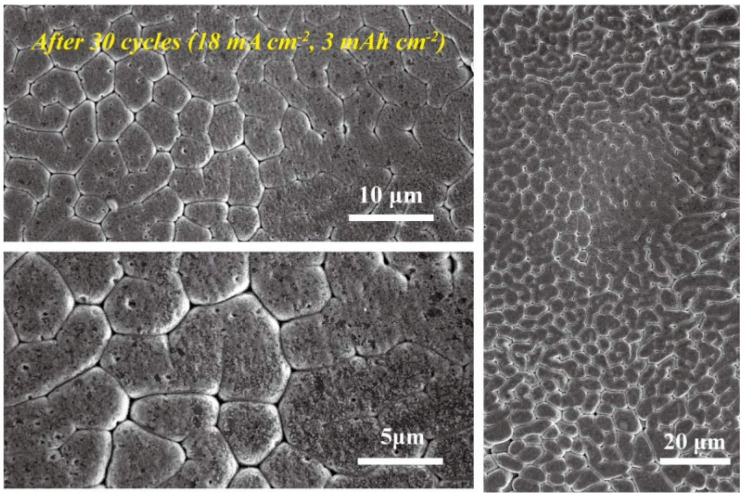

Fig. S9 Morphology SEM images of Li@CFO plating after 30 cycles at 18.0 mA cm^{-2} and 3.0 mA h cm^{-2}

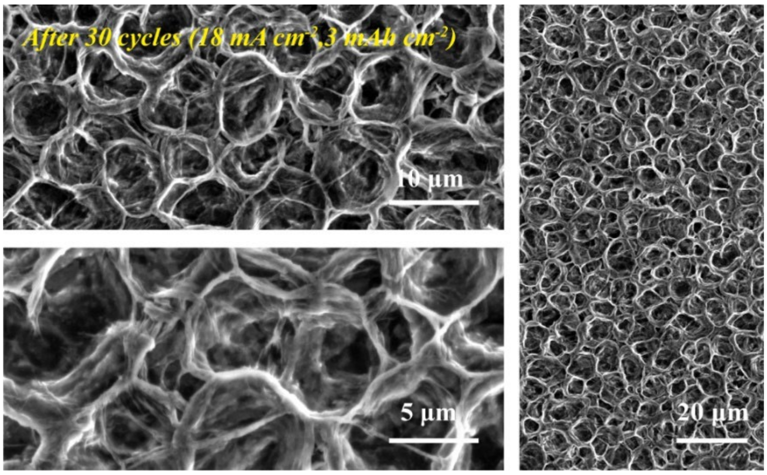

Fig. S10 Morphology SEM images of bare Li plating after 30 cycles at 18.0 mA cm^{-2} and 3.0 mA h cm^{-2}

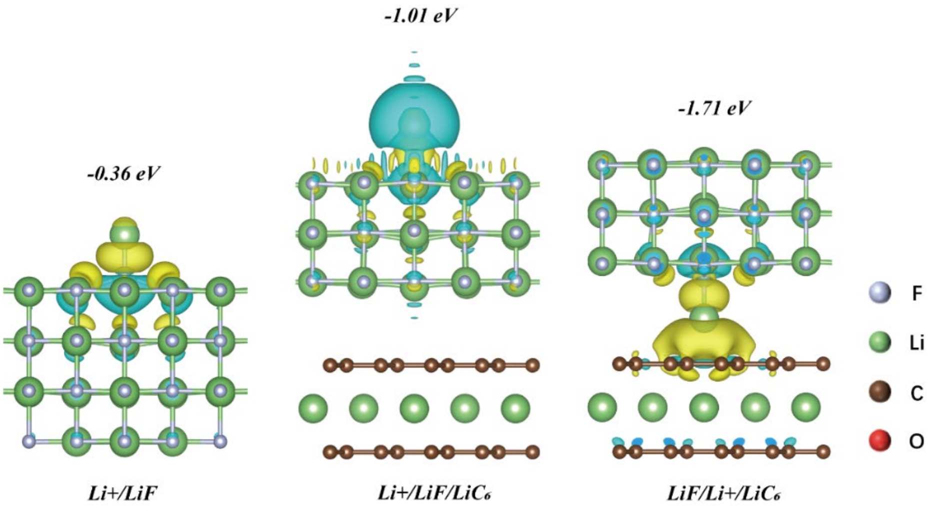

Fig. S11 Charge density difference plot of a Li^+ on the LiF or LiF/LiC_6

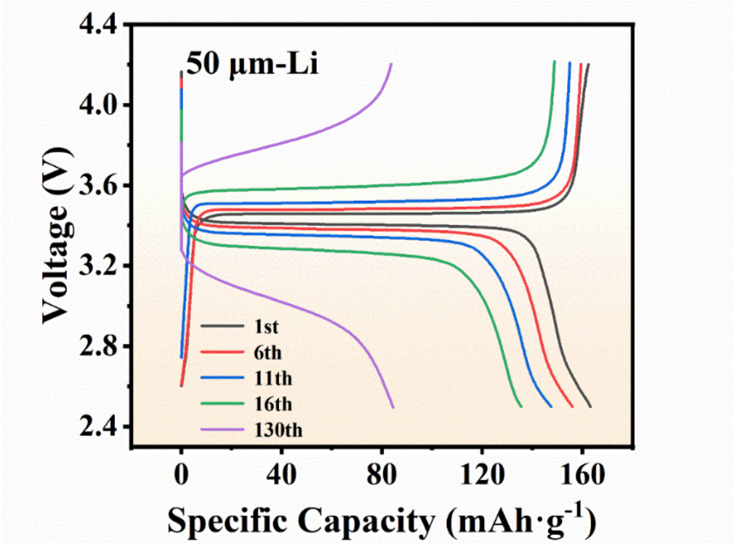

Fig. S12 The corresponding voltage profiles of LFP||Li cells

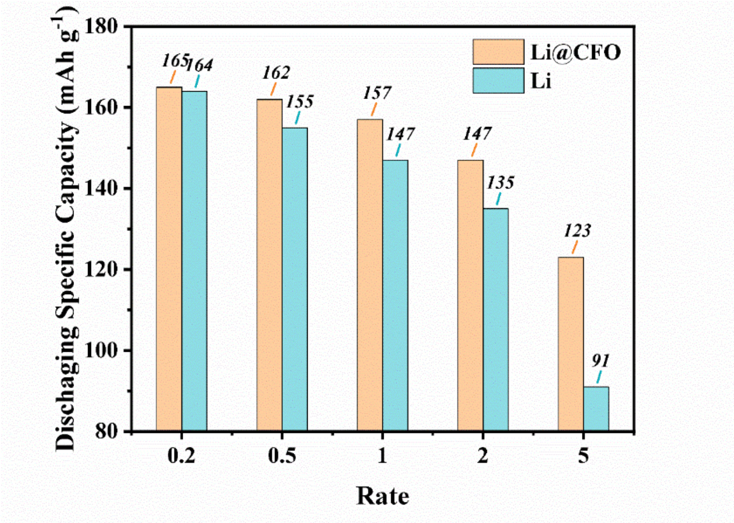

Fig. S13 The rates of LFP||Li cells and LFP||Li@CFO cells

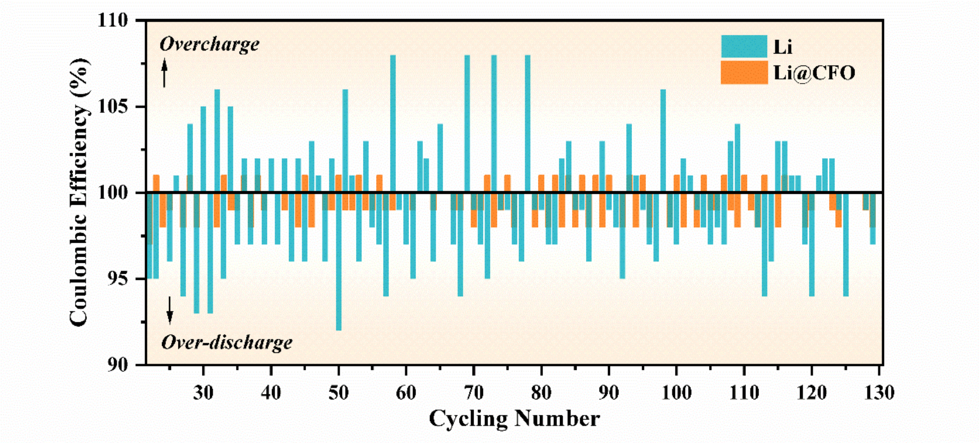

Fig. S14 The CE of LFP||Li cells and LFP||Li@CFO cells
